# Supplementary material for: Comparative Study of Commercial Silica and Sol-Gel-Derived Porous Silica from Cornhusk for Low-Temperature Catalytic Methane Combustion
Source: Nanomaterials (Basel). 2023 Apr 24;13(9):1450. doi: 10.3390/nano13091450 (PMC10180291; doi:10.3390/nano13091450)
Supplement: Supplementary file 1 [file nanomaterials-13-01450-s001.zip › nanomaterials-2351417-supplementary.pdf]

Supplementary Data

Comparative Study of Commercial Silica and Sol−Gel-Derived Porous Silica from Cornhusk for Low-Temperature Catalytic Methane Combustion

Clement Owusu Prempeh [1,2,*], Ingo Hartmann [1,*], Steffi Formann [1], Manfred Eiden [1], Katja Neubauer [3], Hanan Atia [3], Alexander Wotzka [3], Sebastian Wohlrab [3] and Michael Nelles [1,2]

[1] Department of Thermochemical Conversion, DBFZ—Deutsches Biomasseforschungszentrum Gemeinnützige GmbH, Torgauer Straße 116, 04347 Leipzig, Germany; steffi.formann@dbfz.de (S.F.); manfred.eiden@dbfz.de (M.E.); michael.nelles@dbfz.de (M.N.)

[2] Department of Agriculture and Environmental Science, University of Rostock, Justus-von-Liebig-Weg 6, 18059 Rostock, Germany

[3] Leibniz-Institute for Catalysis e.V. (LIKAT), Albert-Einstein-Str. 29a, 18059 Rostock, Germany; katja.neubauer@catalysis.de (K.N.); hanan.atia@catalysis.de (H.A.); alexander.wotzka@catalysis.de (A.W.); sebastian.wohlrab@catalysis.de (S.W.)

* Correspondence: clement.owusuprempeh@dbfz.de (C.O.P.); ingo.hartmann@dbfz.de (I.H.);
Tel.: +49-(0)341-2434-523 (C.O.P.)

Table S1: Bulk elemental compositions measured by ICP-OES of the prepared silica xerogel support (SX) obtained from the cornhusk ash and commercial silica

Elements	Cornhusk support (sol-gel)	Commercial Support
SiO_2	99.69	99.89
P_2O_5	0.15	0.01
SO_3	0.09	0.05
Fe_2O_3	0.03	0.01
MgO	0.02	0.00
Na_2O	0.01	0.00
TiO_2	0.01	0.03

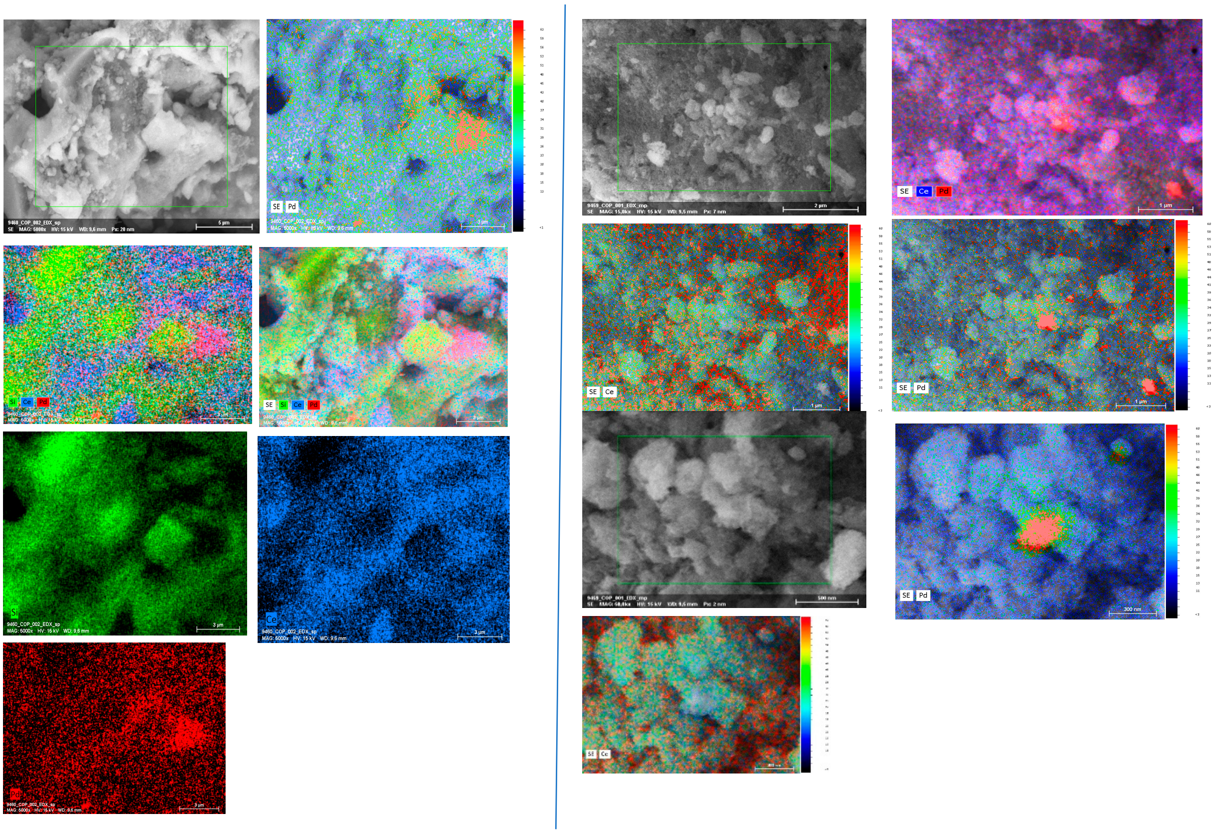

Figure S1. SEM/EDX mappings of $Pd/CeO_2/CHSiO_2$ (left side) and Pd/CeO_2/commercial catalyst (right side)

- EDX mapping at 2 different spots of the catalyst samples
- Clear separation of Pd and Ce can be seen

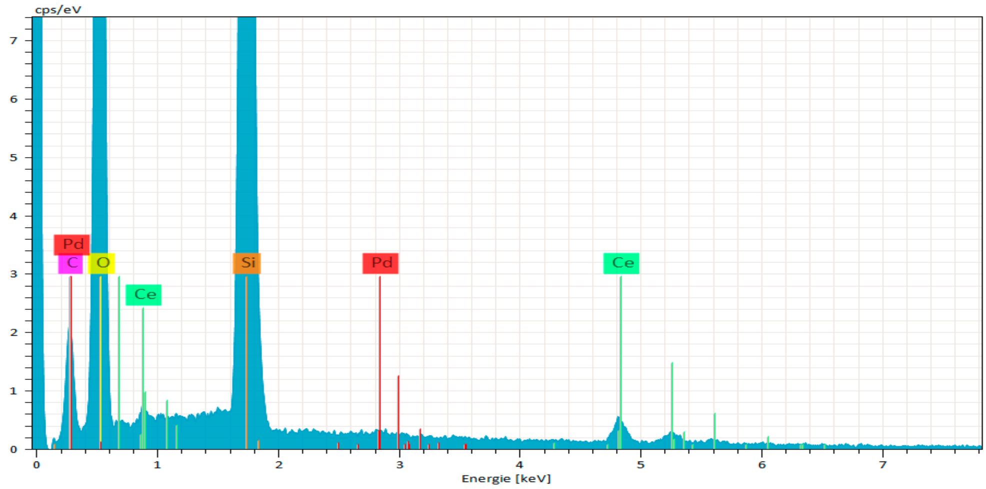

Element	Ord. Z	Net	Wt (%)	Norm mass (%)	Atom (%)	Abs. error (1 sigma)	Rel. error (1 sigma)
**Oxygen**	8	75596	56.28	54.49	66.89	6.47	11.50
**Silicon**	14	144844	37.30	36.12	25.26	1.58	4.23
**Carbon**	6	1613	4.50	4.36	7.13	0.92	20.52
**Ce**	56	3857	4..84	4.69	0.66	0.19	3.94
**Pd**	46	542	0.35	0.34	0.06	0.05	13.13
		Sum	103.27	100	100		

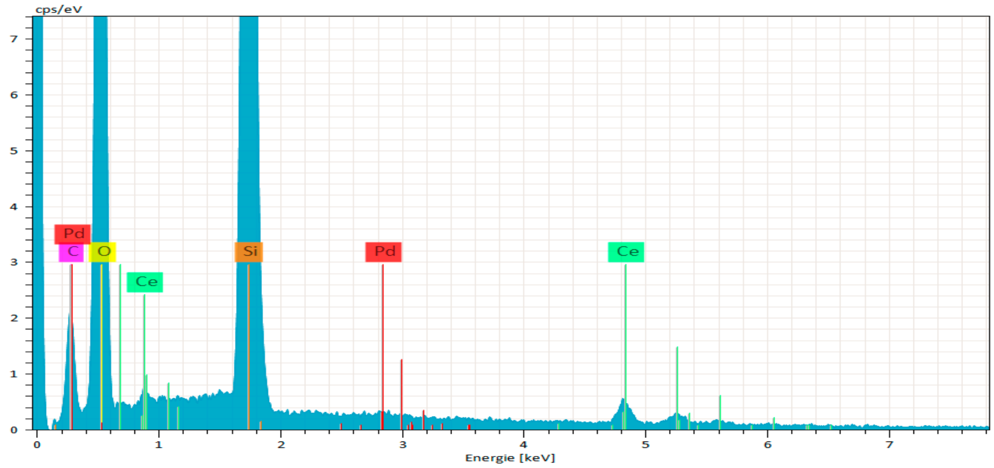

Element	Ord. Z	Net	Wt (%)	Norm mass (%)	Atom (%)	Abs. error (1 sigma)	Rel. error (1 sigma)
**Oxygen**	8	50605	31.30	32.96	55.11	3.71	11.86
**Silicon**	14	106555	33.02	34.78	33.12	1.40	4.24
**Carbon**	6	1462	2.60	2.74	6.10	0.56	21.42
**Ce**	58	27702	27.45	28.91	5.52	0.83	3.04
**Pd**	46	1061	0.58	0.61	0.15	0.05	9.17
		Sum	103.27	100	100		

Figure S2. Details of the two EDX spectra values of Pd/CeO_2/$CHSiO_2$ (top) and Pd/CeO_2/commercial (bottom) catalysts in atomic wt%.

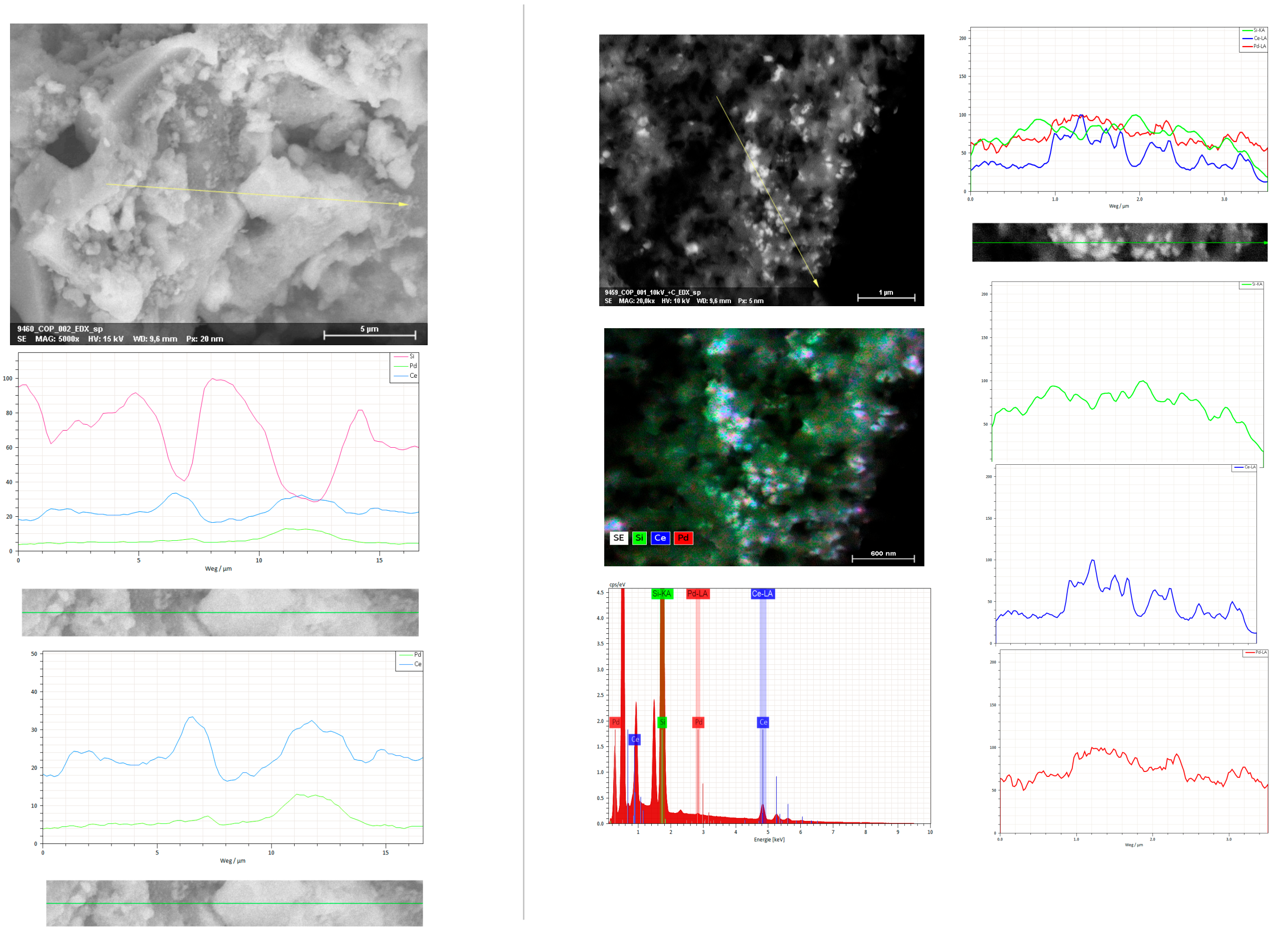

Figure S3. Analysis with BSE (back scattered electrons) detector and line scan for $Pd/CeO_2/CHSiO_2$ (left side) and Pd/CeO_2/commercial (right side) catalysts.

- The Line scan indicates a certain proximity between Ce and Pd
- heavier elements are made more visible by the BSE detector (glow brighter)
- Bright spots indicate areas with Ce and Pd loading

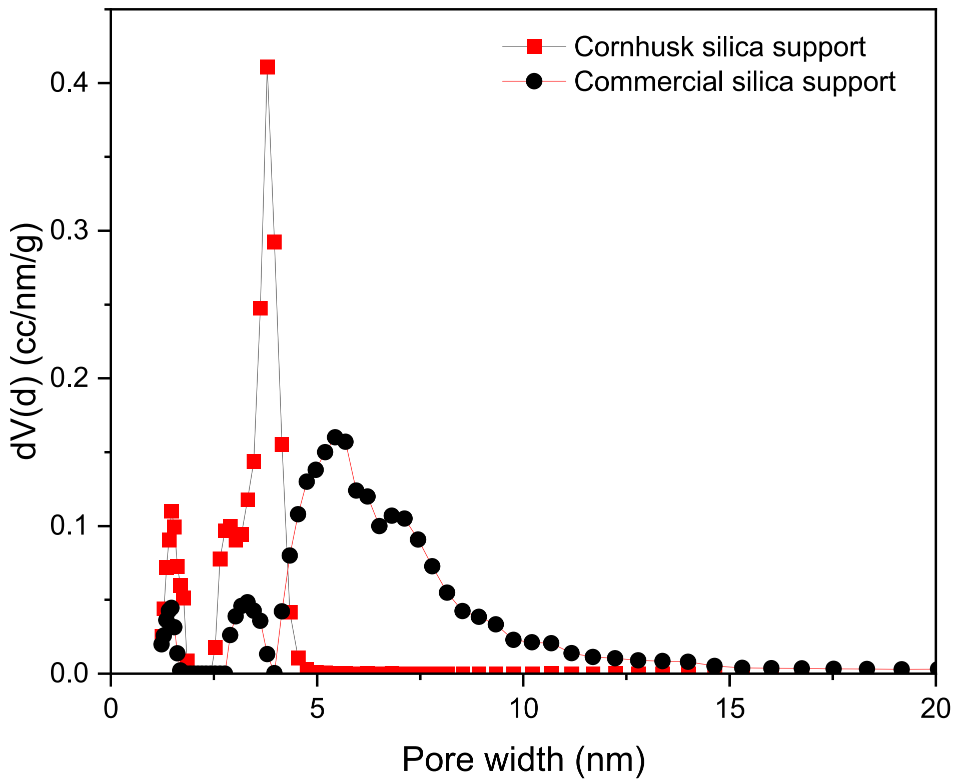

Figure S4. Pore size distribution of cornhusk support and commercial silica support determined by applying a dedicated NLDFT adsorption branch kernel on the adsorption branch of the N_2 (77 K) isotherm

For the cornhusk silica support:
$V_{micro} = 0.123$ cm^3/g
$S_{micro} = 210.841$ m^2/g

For the commercial silica support:
$V_{micro} = 0$ cm^3/g
$S_{micro} = 0$ m^2/g
